# Green Synthesized Titanium Oxide Nanoparticles Promote Salt Tolerance in Soybean

**DOI:** 10.3390/ijms26178309

**Published:** 2025-08-27

**Authors:** Setsuko Komatsu, Pwint Phoo Wai, Tatsuya Takeshita, Yuta Shiraishi

**Affiliations:** 1 Department of Applied Science and Engineering, Fukui University of Technology, Fukui 910-8505, Japan; 2 Department of Applied Chemistry and Food Science, Fukui University of Technology, Fukui 910-8505, Japan; 3 Department of Metrology and Analytical Instrument Development, Otsuka Electronics Co., Ltd., Osaka 573-1132, Japan

**Keywords:** salt stress, soybean, green synthesized titanium oxide nanoparticles, reactive-oxygen species scavenging system

## Abstract

Soybean is rich in protein and functional ingredients, which are in high demand as a food material; however, it is vulnerable to environmental stress. On the other hand, the application of chemically synthesized titanium oxide nanoparticles (TiO_2_ NPs) promoted soybean growth even under salt stress. To further enhance the growth-promoting effect of TiO_2_ NPs on soybeans, they were biologically synthesized using orange peel extract. Root elongation of soybeans suppressed by salt stress was restored to the control level by treatment with green synthesized (GS)-TiO_2_ NPs. To clarify the promoting mechanism in soybean of GS-TiO_2_ NPs under salt stress, immunoblot analysis was performed. The abundance of vacuolar H^+^-ATPase decreased in roots by salt stress was recovered with GS-TiO_2_ NPs. In contrast, the abundance of glutathione reductase increased in roots and hypocotyls by salt stress was recovered with GS-TiO_2_ NPs. Furthermore, hydrogen peroxide production increased in roots with salt stress that was restored by treatment with GS-TiO_2_ NPs. These results suggest that GS-TiO_2_ NPs may restore soybean growth by detoxifying hydrogen peroxide, which increases under salt stress, with upregulating reactive oxygen species scavenging systems.

## 1. Introduction

Agriculture is a core activity in human civilization, constantly facing challenges with the main objective of increasing crop production; to solve this, different strategies and technologies have been used [1]. Currently, metal-based nanoparticles (NPs) have great potential as agricultural inputs to mitigate the negative effects on crops caused by different stresses [2]. Farmers can improve nutrient distribution and avoid waste by focusing on certain areas or individual plants, leading to more resource-efficient and sustainable agricultural practices [3]. Biological synthesis is the process of creating NPs using straightforward, affordable, ecological, and effective technologies. Plant extracts, fungi, yeasts, bacteria, and algae are examples of natural sources, which can be used to biologically synthesize metal and metal oxide NPs [4]. NPs increase crop resilience and yield by assisting plants in adjusting to harsh environmental circumstances such as salt, drought, and heavy metal stress [5]. These reports indicate the necessity for the development of new, innovative stress-mitigating NPs with high efficiency and minimal disadvantages for agriculture.

Among abiotic stresses, salt stress adversely affects plant growth and development through increasing ion toxicity, reducing water uptake, disrupting plant hormones, and elevating reactive-oxygen species (ROS) formation [6]. Metal ions, such as iron, manganese, copper, and zinc, are essential elements for plant growth and development [7]. Under salt stress, the contents of chlorophyll and proline, as well as the activities of ROS-scavenging enzymes, were significantly higher in transgenic *Arabidopsis thaliana* overexpressing MxFRO4 and MxFRO6, which are ferric-reduction oxidase family gene members from *Malus xiaojinensis* [8,9]. The MYB (v-MYB avian myeloblastosis viral oncogene homolog) transcription factor family has numerous members with complex and diverse functions, which play an indispensable role in regulating the response of plants to stress. *FvMYB44* from *Fragaria vesca* performs a role in controlling the response of *A. thaliana* to cold and salt stresses [10]. *FvMYB114* can also promote the expression of genes, such as the genes *AtSOS1/3*, *AtNHX1,* and *AtLEA3* related to salt stress and the genes *AtCCA1*, *AtCOR4*, and *AtCBF1/3* related to cold stress, further improving the tolerance of transgenic plants to these stresses [11]. *MbMYBC1*, isolated from *M. baccata*, has a positive response to low temperature and drought stress. After being introduced into *A*. *thaliana*, the physiological indicators of transgenic plants had corresponding changes under these stresses; furthermore, the activities of ROS scavenging enzymes, electrolyte leakage rate, and the content of proline increased [12]. These findings indicate that MYB transcription factors enhance abiotic tolerance by maintaining ROS homeostasis and transcriptional activation of key ROS-scavenging genes.

Because soybean is a highly salt-sensitive crop, its yield is reduced up to 40% by salinity [13]. Salt stress inhibits root growth and lateral root development, further exacerbating its negative effects [14]. Along with previously mentioned plants, many types of transcriptional factors are active under salt stress in soybeans. The bZIP (basic leucine zipper containing protein) transcription factors, which are one of the largest transcription factor families, are indicated to be associated with various environmental stress tolerances in plants. *GmbZIP15* functions as a negative regulator in response to salt and drought stresses in soybean [15]. The NAC (NAM, ATAF, and CUC) protein family is also one of the largest families of transcription factors in plants. *GmNAC6* is an ideal candidate gene for enhancing salt tolerance of soybean [16]. The DREB (dehydration responsive element binding protein) responds to salt stress through enhanced transcriptional expression and activation of genes involved in plant salinity tolerance. *GmDREB6* is a potential candidate for improving the salt tolerance of soybean [17]. Silicon dioxide NPs with foliar application in sustainable farming improved salinity tolerance by enhancing plant-microbe interactions in soybean [18]. The combined application of plant growth-promoting microbes and silicon-zinc NPs minimized the adverse impact of water stress and soil salinity by maximizing plant growth [19]. The application of titanium oxide (TiO_2_) NPs improved the vegetative growth by increasing pigment and protein contents under salt stress [20,21]. These reports indicate that the use of NPs is beneficial for promoting soybean growth under salt stress.

Among various NPs, TiO_2_ NPs have a wide range of applications; for example, improving the growth and yields of crops, as well as being used in food and cosmetics [22]. TiO_2_ NPs increased the amount of potassium, phosphorus, and nitrogen in wheat under drought stress [23]. TiO_2_ NPs enhanced photosynthetic rate, chlorophyll formation, antioxidant enzyme potential, and ribulose-1,5-bisphosphate carboxylase/oxygenase activity in broad beans under salt stress [24]. TiO_2_ NPs improved germination ratios, water potential, and osmotic potential; increased the carotenoid, total phenolic, flavonoid, soluble sugar, soluble protein, total amino acid, and proline contents; and decreased the malondialdehyde content in wheat under salt stress [25]. TiO_2_ NPs promoted the germination ratio and plant growth of soybeans under salt stress [20,21]. However, the biochemical and molecular mechanisms of TiO_2_ NPs on soybeans under salt stress have not been determined.

In the previous study, chemically synthesized (CS) TiO_2_ NPs promoted soybean growth under salt stress [26]. However, CS TiO_2_ NPs are not suitable for biological applications to crops in soil because of their harmful nature to the environment. Consequently, biological synthesis routes to prepare NPs because this approach is simple, eco-friendly, and cost-effective [27,28]. Of these effects, orange peel as the material for NPs synthesis is the most cost-effective. In this study, to further enhance the growth-promoting effect of TiO_2_ NPs on soybeans, TiO_2_ NPs were biologically synthesized using orange peel. The green synthesized (GS)-TiO_2_ NPs were verified to be NPs by Fourier-transform infrared (FTIR) spectroscopy, ultraviolet-visible reflectance, and multi-angle particle size measurements. Three-day-old soybeans were subjected to GS-TiO_2_ NPs under salt stress for two days, and morphological parameters were measured. To characterize the salt-tolerant mechanism of GS-TiO_2_ NPs in soybeans, immunoblot and polymerase chain reaction (PCR) analyses as well as hydrogen peroxide content measurement were performed, based on the results from proteomic research using commercial CS-TiO_2_ NPs [26].

## 2. Results

### 2.1. GS-TiO_2_ NPs Synthesis and Characterization with FTIR Spectroscopy, Ultraviolet-Visible Reflectance, and Multi-Angle Particle Size Measurement System

**Figure 1:**
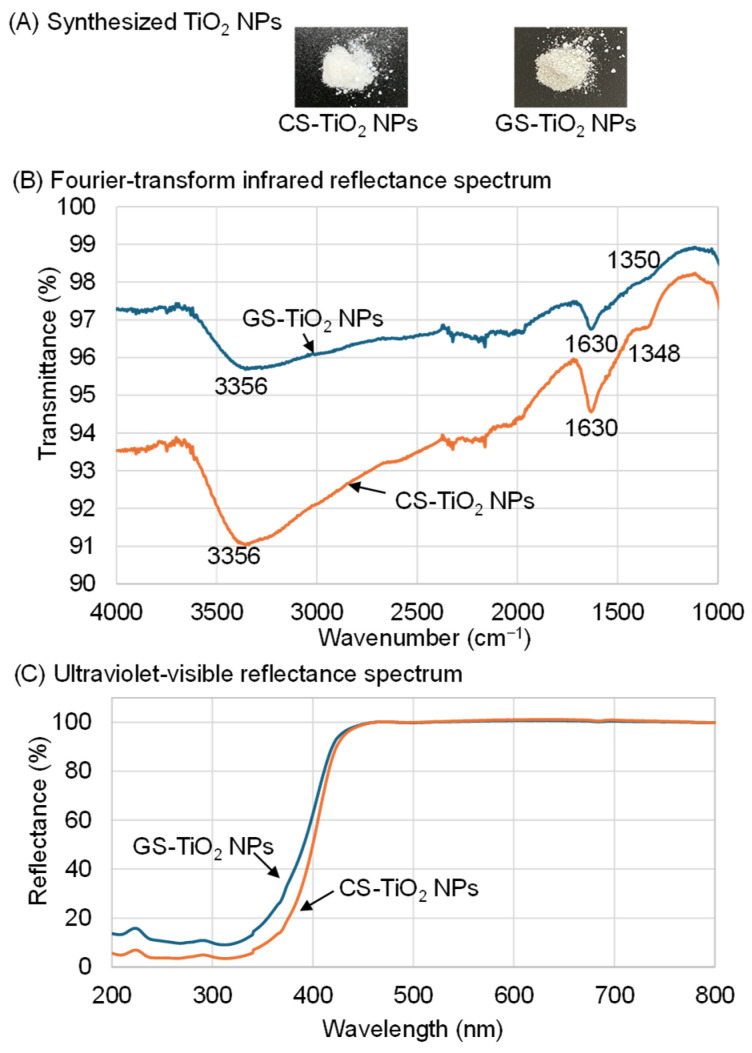
Synthesized TiO_2_ NPs and their spectra analyzed with FTIR spectroscopy and ultraviolet-visible spectroscopy. GS-TiO_2_ NPs were biologically synthesized using orange peel extract. For CS-TiO_2_ NPs, the same procedure was followed to prepare TiO_2_ NPs without orange peel extract. (**A**) Powders of GS-TiO_2_ NPs and CS-TiO_2_ NPs. (**B**) The FTIR spectra of GS-TiO_2_ NPs and CS-TiO_2_ NPs. (**C**) The ultraviolet-visible reflectance spectra of GS-TiO_2_ NPs and CS-TiO_2_ NPs. Blue and orange colors indicate the spectra of GS-TiO_2_ NPs and CS-TiO_2_ NPs, respectively.

To enhance the growth-promoting effect of TiO_2_ NPs on soybeans, GS-TiO_2_ NPs were biologically synthesized using orange-peel extract. For CS-TiO_2_ NPs, the same procedure was followed to prepare TiO_2_ NPs without orange-peel extract (Figure 1A). The CS-TiO_2_ NPs were a pure white powder, while the GS-TiO_2_ NPs were slightly yellow (Figure 1A).

To reveal the formation of the TiO_2_ NPs using the biological synthesis method, attenuated total reflection (ATR)-FTIR spectra were measured (Figure 1B). The infrared spectrum of the GS-TiO_2_ NPs showed a peak at 3356, 1630, and 1350 cm^−1^ (Figure 1B, blue color). In the case of CS-TiO_2_ NPs, the infrared spectrum showed a peak at 3356, 1630, and 1348 cm^−1^ (Figure 1B, orange color).

The ultraviolet-visible reflectance spectra of GS-TiO_2_ NPs and CS-TiO_2_ NPs were measured (Figure 1C). The absorption band edge observed at approximately 400 nm and the strong reflection in the ultraviolet region are consistent with the optical properties of typical TiO_2_ NPs.

**Figure 2:**
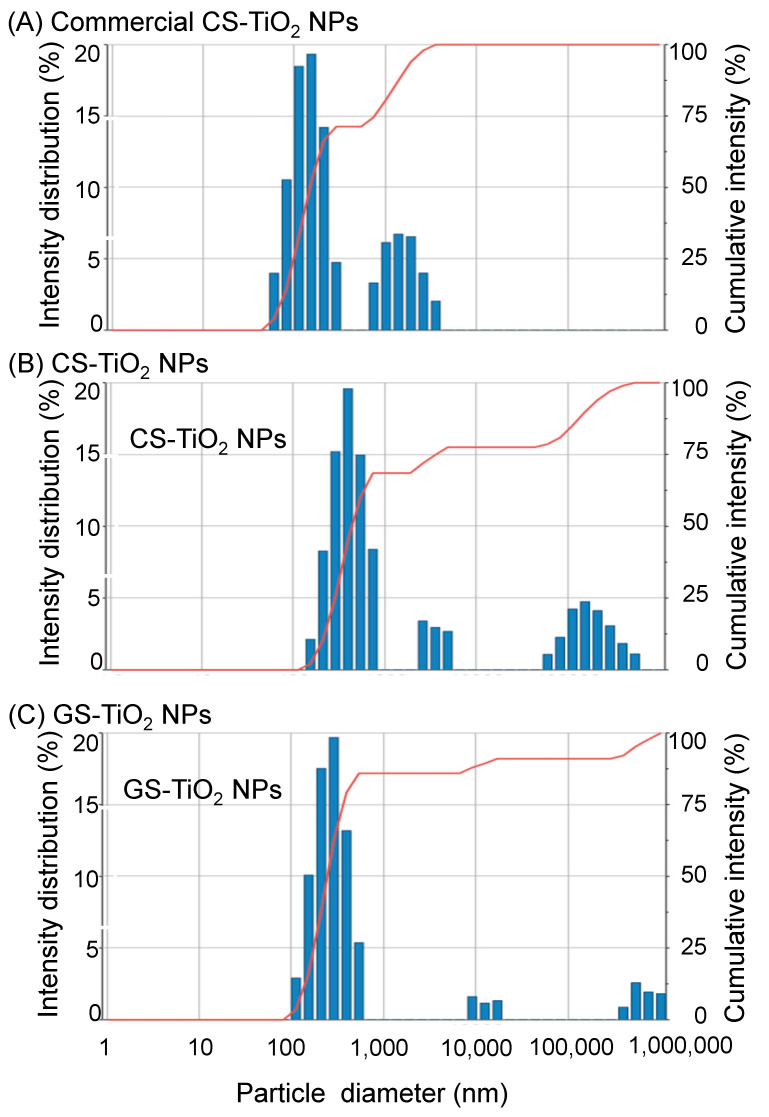
The particle sizes of synthesized TiO_2_ NPs. The particle sizes of TiO_2_ NPs were analyzed using zeta-potential and particle size analyzer. The particle sizes of commercial CS-TiO_2_ NPs (**A**), CS-TiO_2_ NPs (**B**), and GS-TiO_2_ NPs (**C**) were analyzed with solution. Blue column shows the intensity distribution (%) and red line shows cumulative intensity (%).

The particle sizes of TiO_2_ NPs were analyzed using zeta-potential and a particle size analyzer (Figure 2). The average particle sizes of commercial CS-TiO_2_ NPs, CS-TiO_2_ NPs, and GS-TiO_2_ NPs were 145.7 nm (Figure 2A), 410.4 nm (Figure 2B), and 308.6 nm (Figure 2C), respectively.

### 2.2. Morphological Analysis of Soybean Treated with TiO_2_ NPs Under Salt Stress

**Figure 3:**
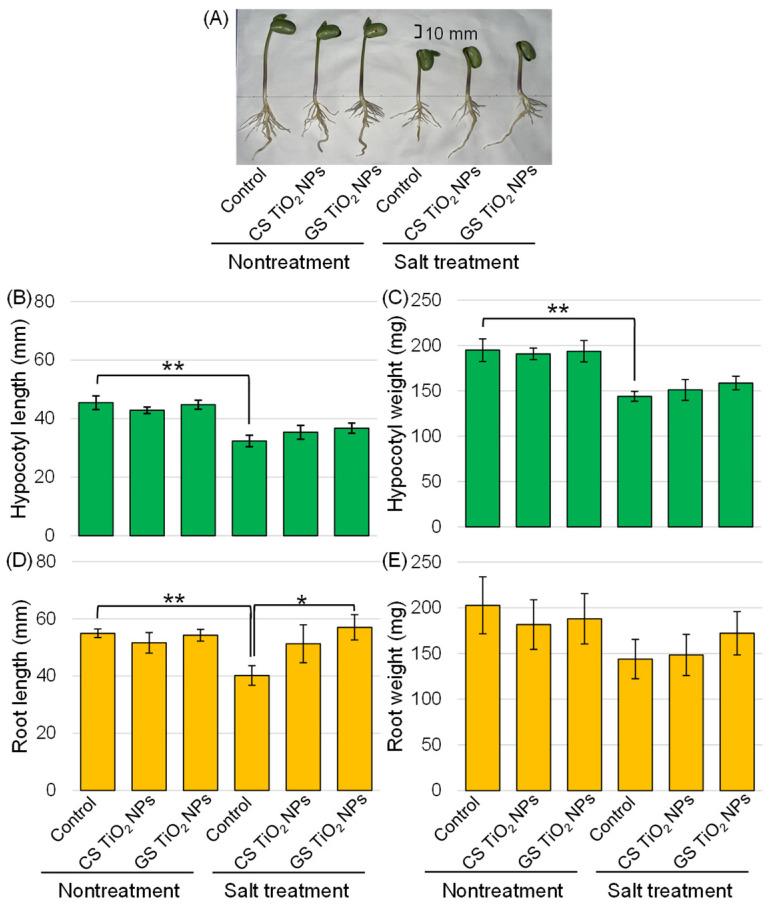
Morphological analysis of soybean treated with TiO_2_ NPs under salt stress. Soybean seeds were sown and grown for three days. Seedlings were treated with or without 40 μg/mL TiO_2_ NPs with or without 150 mM NaCl for two days. Before morphological analysis, a photograph was taken (**A**). The bar in the picture indicates 10 mm. As morphological parameters, hypocotyl length (green color) (**B**), hypocotyl fresh weight (green color) (**C**), main root length (orange color) (**D**), and total root fresh weight (orange color) (**E**) were measured at 5 days after sowing. Data are presented as the mean ± SD from 3 independent biological replicates. The statistical significance of two groups was evaluated by the Student’s *t*-test. A *p*-value of less than 0.05 was considered statistically significant (**, *p* < 0.01; *, *p* < 0.05).

Morphological analysis was conducted to investigate the effects of GS-TiO_2_ NPs on soybeans under salt stress. Three-day-old seedlings were treated with or without 40 μg/mL GS- and CS-TiO_2_ NPs with or without 150 mM NaCl for 2 days. Six types of treatments were performed: control, CS-TiO_2_ NPs, GS-TiO_2_ NPs, salt, salt + CS-TiO_2_ NPs, and salt + GS-TiO_2_ NPs treatments (Figure 3A). Morphological parameters such as hypocotyl length, hypocotyl fresh weight, main root length, and total root fresh weight were measured (Figure 3B–E). Although the length and weight of hypocotyl were suppressed by salt stress, they could not recover with the treatment of GS-TiO_2_ NPs (Figure 3B,C). Soybean-root length was suppressed by salt stress and recovered with GS-TiO_2_ NPs compared with the control; however, root weight did not change with any treatments (Figure 3D,E).

### 2.3. Analysis of Chlorophylls a and b Contents

**Figure 4:**
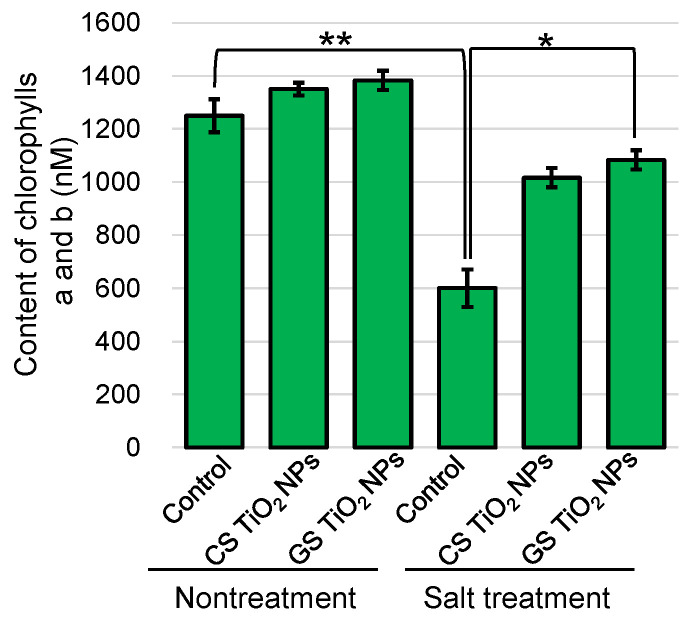
The contents of chlorophylls *a* and *b* in soybean leaf. Six treatments were performed: control, CS-TiO_2_ NPs, GS-TiO_2_ NPs, salt, CS-TiO_2_ NPs + salt, and GS-TiO_2_ NPs + salt. Chlorophylls *a* and *b* extracted from soybean leaf were measured. Data analysis and statistical calculations are the same as in Figure 3 (**, *p* < 0.01; *, *p* < 0.05).

To understand the photosynthetic performance, chlorophylls *a* and *b* have been measured using the top leaf (Figure 4). The contents of chlorophylls *a* and *b* significantly decreased under salt stress; however, they recovered by additional GS TiO_2_ NPs, even if it was under stress (Figure 4). These results indicated that photosynthesis was improved by additional GS TiO_2_ NPs, even if it was under stress, although hypocotyl length and weight could not recover with the treatment of GS-TiO_2_ NPs.

### 2.4. Analysis of Hydrogen Peroxide Content in Soybean Root Treated with TiO_2_ NPs Under Salt Stress

**Figure 5:**
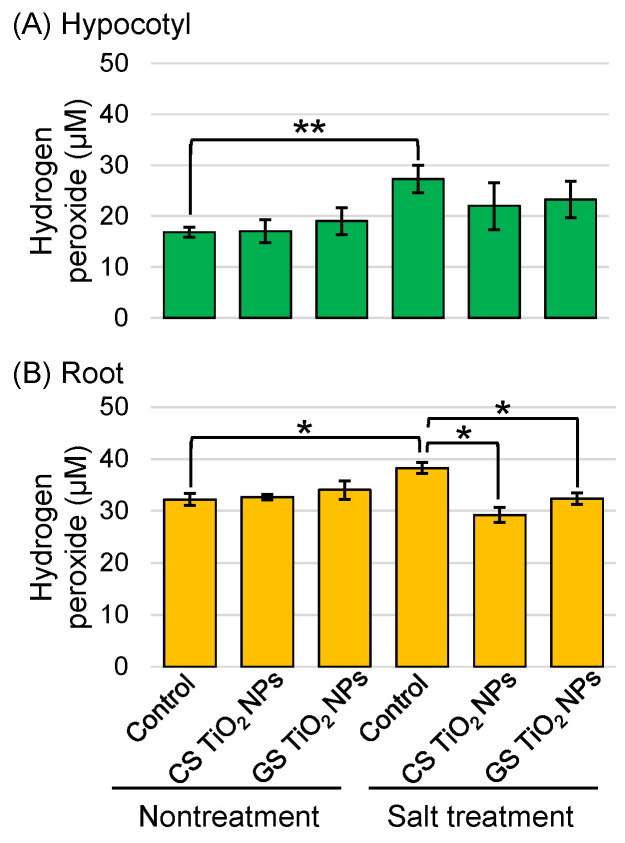
Analysis of hydrogen-peroxide content in soybean treated with TiO_2_ NPs under salt stress. Six treatments were performed: control, CS-TiO_2_ NPs, GS-TiO_2_ NPs, salt, CS-TiO_2_ NPs + salt, and GS-TiO_2_ NPs + salt. The extracts from hypocotyl (**A**) and root (**B**) of soybean were reacted with the addition of working solution. Data analysis and statistical calculations are the same as in Figure 3 (**, *p* < 0.01; *, *p* < 0.05).

To understand the accumulation of ROS by TiO_2_ NPs in soybean plants under salt stress, the amount of hydrogen peroxide was measured based on the results from proteomic research using commercial CS-TiO_2_ NPs [17]. The amount of hydrogen peroxide increased in the root and hypocotyl with salt stress compared to the control (Figure 5A,B), and it was restored by treatment with GS-TiO_2_ NPs and CS-TiO_2_ NPs in the root (Figure 5B).

### 2.5. Immunoblot Analysis of Proteins in Soybean Treated with TiO_2_ NPs Under Salt Stress

To better uncover the differential abundant proteins treated with TiO_2_ NPs, immunoblot analysis was performed. Proteins extracted from the root and hypocotyl of soybean were separated on sodium dodecyl sulfate (SDS)-polyacrylamide gel by electrophoresis and transferred to polyvinylidene-difluoride (PVDF) membrane. The membranes were cross-reacted with superoxide dismutase, peroxiredoxin, ascorbate peroxidase, glutathione reductase, and vacuolar H^+^-ATPase (V-ATPase) antibodies. A staining pattern with Coomassie brilliant blue was used as a loading control (Figure S1). The integrated densities of bands were calculated using ImageJ software with triplicated immunoblot results (Figures S2–S6).

**Figure 6:**
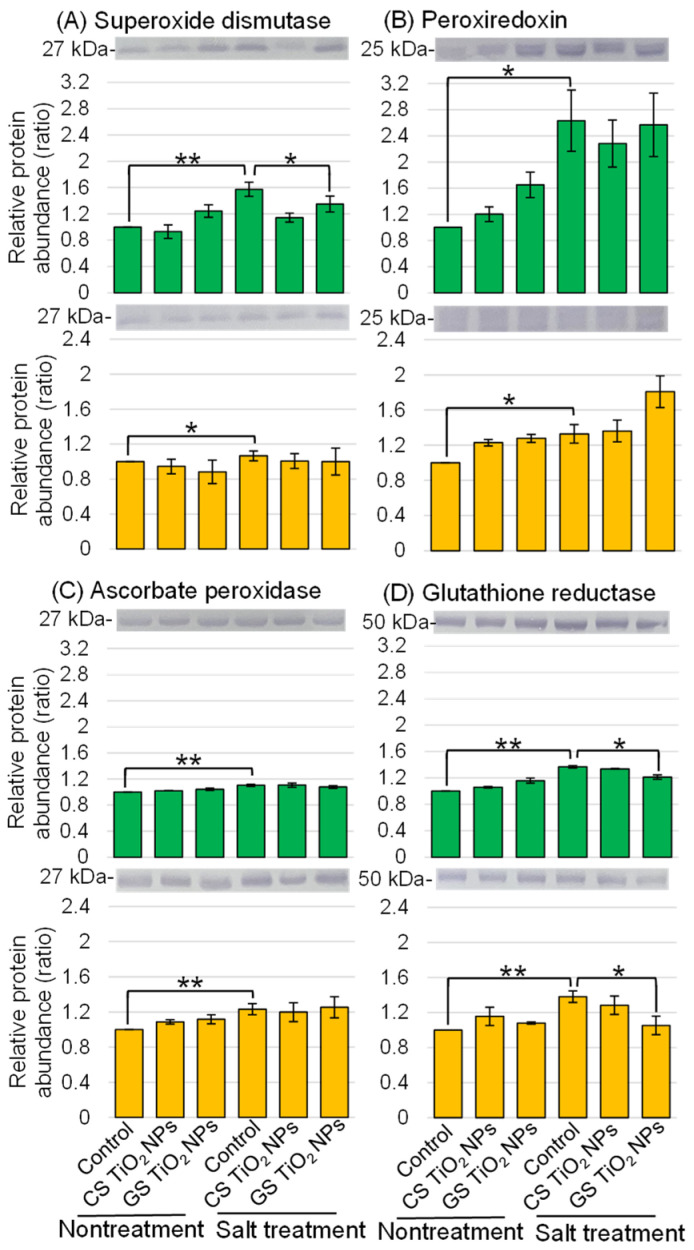
Immunoblot analysis of proteins involved in soybean treated with TiO_2_ NPs under salt stress. Six treatments were performed: control, CS-TiO_2_ NPs, GS-TiO_2_ NPs, salt, CS-TiO_2_ NPs + salt, and GS-TiO_2_ NPs + salt. Proteins extracted from root and hypocotyl of soybean were separated on SDS-polyacrylamide gel by electrophoresis. Proteins were transferred onto PVDF membranes. The membranes were cross-reacted with anti-superoxide dismutase, peroxiredoxin, ascorbate peroxidase, and glutathione reductase antibodies. The integrated densities of the bands were calculated using ImageJ software. Data analysis and statistical calculations are the same as in Figure 3 (**, *p* < 0.01; *, *p* < 0.05).

**Figure 7:**
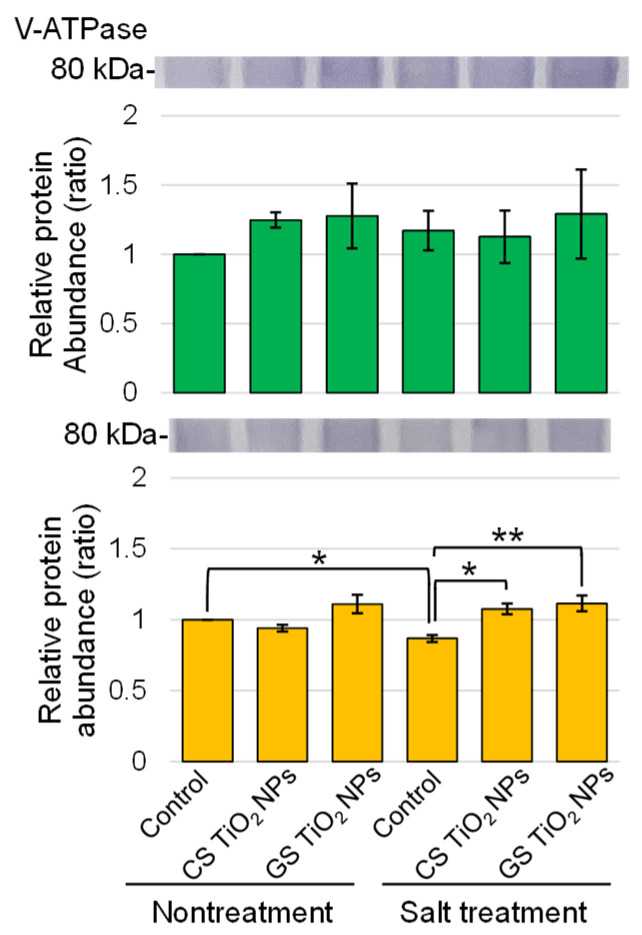
Immunoblot analysis of proteins involved in soybean treated with TiO_2_ NPs under salt stress. Six treatments were performed: control, CS-TiO_2_ NPs, GS-TiO_2_ NPs, salt, CS-TiO_2_ NPs + salt, and GS-TiO_2_ NPs + salt. Proteins extracted from root and hypocotyl of soybean were separated on SDS-polyacrylamide gel by electrophoresis. Proteins were transferred onto PVDF membranes. The membranes were cross-reacted with anti-V ATPase antibody. The integrated densities of the bands were calculated using ImageJ software. Data analysis and statistical calculations are the same as in Figure 3 (**, *p* < 0.01; *, *p* < 0.05).

Superoxide dismutase, peroxiredoxin, ascorbate peroxidase, and glutathione reductase were selected as the proteins related to the ROS scavenging system (Figure 6). The abundances of the proteins related to the ROS scavenging system increased in soybean root and hypocotyl under salt stress (Figure 6A–D). Among them, the abundance of glutathione reductase increased in roots and hypocotyls under salt stress was recovered by additional treatment with GS-TiO_2_ NPs (Figure 6D). In contrast, the abundance of V-ATPase decreased in roots under salt stress and was recovered by application with GS-TiO_2_ NPs even under stress (Figure 7).

### 2.6. Analysis of Ascorbate Peroxidase Activity

**Figure 8:**
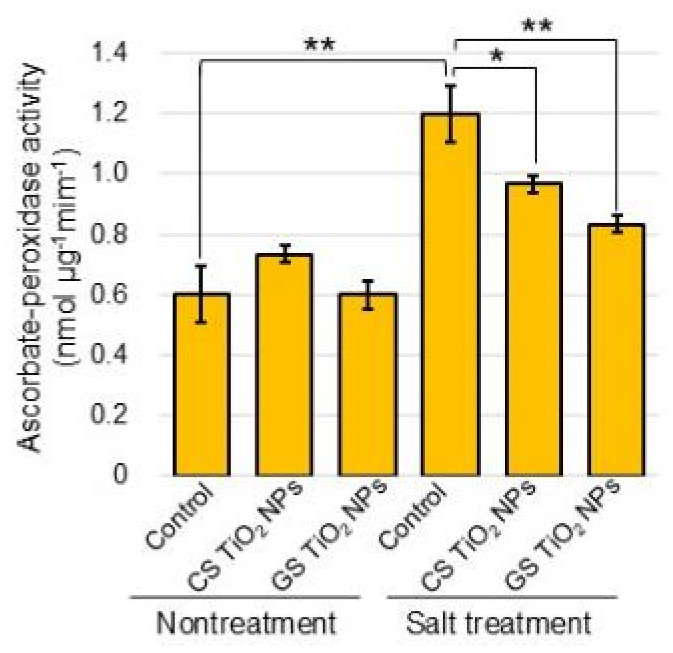
The ascorbate peroxidase activity of extracts from soybean root. Six treatments were performed: control, CS-TiO_2_ NPs, GS-TiO_2_ NPs, salt, CS-TiO_2_ NPs + salt, and GS-TiO_2_ NPs + salt. Using extracts of soybean root, ascorbate peroxidase activity was determined by monitoring the oxidation of ascorbic acid at 290 nm. Data analysis and statistical calculations are the same as in Figure 3 (**, *p* < 0.01; *, *p* < 0.05).

To understand the quantitative enzyme activity, ascorbate-peroxidase activity was measured (Figure 8). Ascorbate peroxidase activity was upregulated in roots under salt stress; however, they were recovered with the application of GS TiO_2_ NPs and CS TiO_2_ NPs (Figure 8). Although immunoblot analysis could not detect the change in accumulation by GS TiO_2_ NPs application, quantitative enzyme activity assay could detect those changes.

### 2.7. PCR Analysis of the Gene Encoding Bet v1 in Soybean with Application of TiO_2_ NPs Under Salt Stress

**Figure 9:**
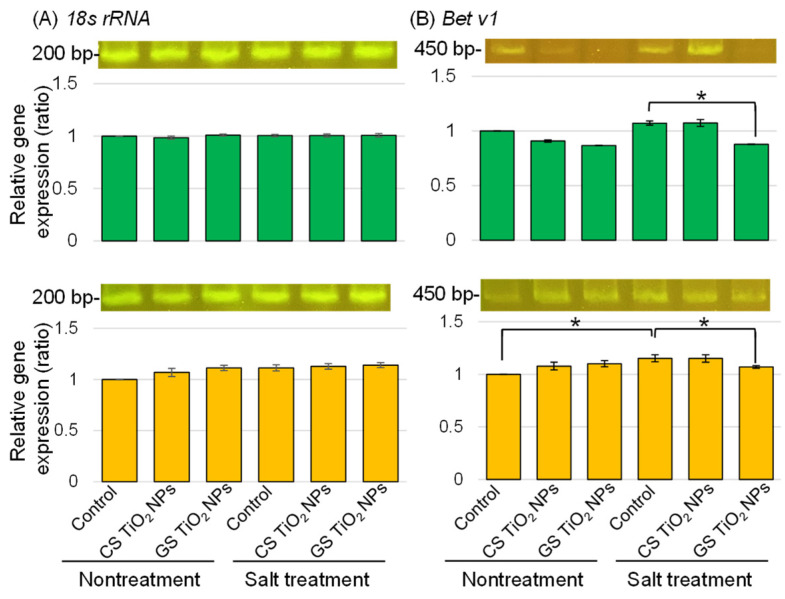
The expression of gene-encoding bet v1 involved in soybean treated with TiO_2_ NPs under salt stress. Six treatments were performed: control, CS-TiO_2_ NPs, GS-TiO_2_ NPs, salt, CS-TiO_2_ NPs + salt, and GS-TiO_2_ NPs + salt. After isolating total RNA from root and hypocotyl of soybean, *18S rRNA* (**A**)-specific and *bet v1* (**B**)-specific oligonucleotides were amplified using PCR. *18S rRNA* was used as an internal control. After agarose-gel electrophoresis, the integrated densities of the bands were calculated using ImageJ software. Data analysis and statistical calculations are the same as in Figure 3 (* *p* < 0.05).

*Bet v1*-specific oligonucleotide was used to amplify transcripts of the total RNA isolated from soybean root and hypocotyl (Figure 9). The expression level of *18S rRNA* was used as an internal control (Figure 9A). The expression of *bet v1* was upregulated by salt stress; however, this expression recovered to control level with GS-TiO_2_ NPs, even with stress (Figure 9B).

## 3. Discussion

### 3.1. GS-TiO_2_ NPs Promote Soybean Root Under Salt Stress

The application of commercial CS-TiO_2_ NPs promoted soybean growth under salt stress [17]. To further enhance the growth-promoting effect of TiO_2_ NPs on soybeans, GS-TiO_2_ NPs were biologically synthesized using orange peel extract [29]. The liquid extract of orange peel was slowly added to the TiCl_4_ with stirring, which resulted in a change from a milky off-white to a slightly yellow color (Figure 1). During the biosynthesis process, the colloidal solution turns from white to yellowish gray, which indicates the formation of TiO_2_ NPs [30]. The white color dispersion shows the formation of TiO_2_ NPs during the chemical process [30].Visual observation of color change was considered as an initial sign of synthesis of TiO_2_ NPs, and this was in with the previous reports, although their colors were pinkish brown [21,31] and yellowish gray [30]. Since previous materials were aloe leaves, these color differences indicate biological sources.

To reveal the formation of TiO_2_ NPs using the biological synthesis method, ATR-FTIR spectra of GS-TiO_2_ NPs were measured (Figure 1). The peak at 3356 cm^−1^ was assigned to the stretching vibrations of the hydroxyl group (-OH) [32]. The peaks at 1630 and 1350 cm^−1^ were assigned to the -OH bending of surface-adsorbed water and Ti-O-Ti stretching vibrations, respectively [30]. Likewise, these peaks were observed in the infrared spectrum of CS-TiO_2_ NPs. Therefore, it can be concluded that TiO_2_ NPs were successfully formed using a biological synthesis method.

The ultraviolet-visible reflectance spectra of GS- and CS-TiO_2_ NPs were measured, presenting the absorption band edge observed at approximately 400 nm and the strong reflection in the ultraviolet region (Figure 1). The sharp absorption peak corresponds to the change in the crystalline phase and the average crystalline size [33], meaning that the investigated nanomaterial is applicable for catalytic application [34]. The sharp absorbance peak around the 385–400 nm region indicates the formation of TiO_2_ NPs [30]. The reflectance spectra of TiO_2_ NPs were well matched with the previous reports [35]. This result supported the formation of TiO_2_ NPs, as confirmed by FTIR spectroscopy. The difference in reflectance in the ultraviolet region between GS- and CS-TiO_2_ NPs may be attributed to the difference in surface morphology and/or particle size.

The particle sizes of TiO_2_ NPs were analyzed, and the average particle sizes of commercial CS-TiO_2_ NPs, CS-TiO_2_ NPs, and GS-TiO_2_ NPs were 145.7 nm, 410.4 nm, and 308.6 nm, respectively (Figure 2). Although the size of commercial CS-TiO_2_ NPs is 25 nm, in water they enlarged to 145.7 nm. For example, the transmission electron microscopy images of zirconium sulfide (ZrS_2_) NPs demonstrated that ZrS_2_ NPs agglomerated to form nanoclusters [36]. The platinum NPs are unstable because of agglomeration and Ostwald ripening [37]. Previous findings with this result suggest that TiO_2_ NPs synthesized in this study might be agglomerated. The average particle sizes for GS- and CS-TiO_2_ NPs support the difference in the reflectance of the ultraviolet-visible reflectance spectra. GS-TiO_2_ NPs with small particle sizes are expected to be more effective on soybeans than CS-TiO_2_ NPs.

Growth of soybean seedlings was adversely affected by salt stress with 200 mM NaCl [21]. Similarly, the hypocotyl and root of soybean were suppressed by 150 mM NaCl [26]. The decrease in growth under salt stress is due to osmotic and ion toxicity. Additionally, the uptake of nutrients and water is greatly affected under salt stress through the reduced metabolic activity of seedlings [38]. In this study, soybean growth was significantly suppressed by salt stress, in line with previous reports (Figure 3). However, soybean root length suppressed by salt stress recovered with GS-TiO_2_ NPs compared with CS-TiO_2_ NPs (Figure 3). Furthermore, it is reported that TiO_2_ NPs enhanced seedling emergence, vigor, and tolerance in soybeans under salinity stress [21,26]. In the case of commercial TiO_2_ NPs, which are CS-TiO_2_ NPs, their effects were recognized on the hypocotyl of soybean [26]. These results suggest that the smaller sizes of NPs are more effective aboveground, even if they are CS-TiO_2_ NPs.

### 3.2. ROS-Scavenging System Relates to Salt-Tolerant Mechanism in Soybean with GS-TiO_2_ NPs

Salt stress affects plants by imposing various complications such as ion toxicity, osmotic stress, nutritional deficiency, and genotoxicity, resulting in ROS overproduction and oxidative stress [39]. Hydrogen peroxide content and antioxidant enzyme activity increased by heightening NaCl concentrations [21]. In the present study, hydrogen-peroxide content and ascorbate peroxidase activity showed the same results (Figure 5 and Figure 8). In plants treated with TiO_2_ NPs, a decrease in hydrogen peroxide level is due to the accumulated level of superoxide dismutase and peroxidase [40,41]. The amount of hydrogen peroxide and the abundance of glutathione reductase increased with salt stress; however, they did not change with the addition of commercial CS-TiO_2_ NPs [17]. In this study, the amount of hydrogen peroxide and the abundance of glutathione reductase increased in soybean roots with salt stress were recovered with additional GS-TiO_2_ NPs, even if under stress (Figure 5 and Figure 6). It is suggested that GS-TiO_2_ NPs, which could directly alleviate oxidative stress by reducing hydrogen peroxide, improve soybean growth even if under salt stress. However, commercial CS-TiO_2_ NPs improve soybean growth through by increasing the glutathione reductase and decreasing hydrogen peroxide.

### 3.3. Soybean Tonoplast Functions with Application of GS-TiO_2_ NPs Under Salt Stress

Two types of proton pumps, which are vacuolar H^+^-pyrophosphatase and V-ATPase, provide energy for transporting ions across the tonoplast in *A*. *thaliana* [42]. One of these, V-ATPase, which is highly conserved across species, consists of a transmembrane V0 complex and a cytoplasmic V1 complex [43]. V-ATPase generates the proton gradient and membrane potential required for proton-coupled anion transport into compartments, including the large central vacuole [44]. V-ATPase plays a critical role in cytosolic ion homeostasis, which affects cell growth [45]. For example, V-ATPase B subunit 3 was required for cell growth and ionic homeostasis in *A*. *thaliana* [46]. The basic helix-loop-helix transcription factor 62/V-ATPase B subunit 1 module played a positive role in salt tolerance by maintaining intracellular ion and ROS homeostasis in pear [47]. The exposure of plants to silver NPs affects the expression of genes and accumulation of proteins involved in maintaining cellular electrochemical gradient through H^+^-ATPase and V-ATPase, as well as redox potential [48]. In this study, the abundance of V-ATPase decreased in roots under salt stress and was restored by additional treatment with GS- and CS-TiO_2_ NPs (Figure 7). The present result and previous findings suggest that salt stress suppresses the function of the tonoplast, but the addition of TiO_2_ NPs restores its function and relieves excessive salt stress. Moreover, this feature is possessed by both chemically and biologically synthesized NPs.

### 3.4. Soybean Bet v1 Functions with Application of GS-TiO_2_ NPs Under Salt Stress

The pathogenesis-related 10 (PR10) protein family, which is known as the bet v1 family, includes classical PR10 proteins, major latex proteins (MLPs), cytokinin receptors, and plant polyketide cyclase-like proteins [49]. One potential component of the adaptation mechanisms is the PR10-protein family, which plays an important role in enhancing tolerance to both biotic and abiotic stresses [50]. Bet v1 like PR10 protein, was identified to interact with chitinases and enhanced defense responses against anthracnose, highlighting its role in biotic stress tolerance [51]. On the other hand, MLPs were involved in drought and salt tolerance through mediating phytohormone signaling pathways [52]. For example, the overexpression of *GhMLP28* from cotton in *A*. *thaliana* improved salt–stress tolerance [53]. Additionally, its expression in *Nicotiana tabacum* enhanced resistance to *Verticillium dahliae* infection [54], overexpression of *AtMLP43* enhanced drought tolerance through the abscisic acid signaling pathway [55,56]. The overexpression of *PvMLP19* was associated with delayed seed germination but promoted root development under osmotic and salt stress [50]. In this study, the expression of *bet v1* was upregulated by salt stress in the root; however, this expression recovered to the control level with GS-TiO_2_ NPs, even with stress (Figure 9). These results indicate that GS-TiO_2_ NPs do not induce salt-stress tolerance by increasing bet v1, because bet v1 was down-regulated by additional TiO_2_ NPs when salt stress was present. Ti may bind to chloride ions from NaCl, which is used as a salt stress-induced agent, and decrease the amount of NaCl, which is reducing the expression of TiO_2_ NPs on bet v1.

## 4. Materials and Methods

### 4.1. Preparation of Orange Peel Extract and Synthesis of TiO_2_ NPs

Pieces (20 g) of fresh orange peels were placed in a beaker with 100 mL of deionized water and allowed to boil for 2 h. The extract was filtered with Whatman filter paper (Cytiva, Tokyo, Japan) to remove the impurities. Then, 100 mL of 0.1 M TiCl_4_ aqueous solution was prepared by adding Ti (IV) chloride solution (16.0–17.0% (as Ti), 29.0–33.0% (as Cl); Wako, Osaka, Japan) to deionized water. In addition, the prepared 10 mL of extract was slowly added under magnetic stirring. Subsequently, 1 M NaOH was added to adjust the pH to 7. Precipitates, which consider the TiO_2_ NPs formed immediately after the addition of NaOH, were collected using Whatman’s filter paper. The synthesized TiO_2_ NPs were washed 10 times with deionized water to remove the impurities, dried at 100 °C for 10 h, and calcined at 500 °C for 4 h using a muffle furnace. The final powder was named GS-TiO_2_ NPs. The same procedure was followed to prepare TiO_2_ NPs without orange-peel extract by chemical method, which was named CS-TiO_2_ NPs. Finally, the samples were prepared by chemical and green synthesis methods [29].

### 4.2. Analyses of TiO_2_ NPs with FTIR Spectroscopy, Ultraviolet-Visible Spectroscopy, and Multi-Angle Particle Size Measurement System

GS-TiO_2_ NPs were characterized using different techniques using FTIR spectroscopy (Spectrum One FTIR Spectrometer; PerkimElmer, Shelton, CT, USA) with a universal ATR sampling accessory (diamond/KRS-5), ultraviolet-visible spectroscopy (V-650 Spectrophotometer; JASCO, Tokyo, Japan) with an integrating sphere unit (ISV-922), and a zeta-potential and particle size analyzer (ELSZneo; Otsuka, Osaka, Japan). The baseline of the ultraviolet-visible reflectance spectra was subtracted using Origin 2024b (64-bit) software (Light Stone, Tokyo, Japan). Water was used as the solvent for particle-size analysis. To remove the coarse particles, the solution was filtered twice with a 5 μm filter before measurement.

### 4.3. Plant Materials and Treatment

Seeds of soybean (*Glycine max* L. cultivar Enrei) were sown on silica sand. After 3 days of sowing, soybean was treated with or without 40 μg/mL GS-TiO_2_ NPs or CS-TiO_2_ NPs and with or without 150 mM NaCl for 2 days. Seedlings were maintained at 25 °C in a growth chamber illuminated with white-fluorescent light (200 μmol m^−2^ s^−1^, 16 h light period/day) and 70% relative humidity. Main-root length, total root-fresh weight, hypocotyl length, and hypocotyl-fresh weight were measured as morphological parameters. For other experiments, the root and hypocotyl were separately collected. Three independent experiments were performed as biological triplicates for all experiments. As independent biological replicates, soybeans were sown on different days.

### 4.4. Measurement of Chlorophylls a and b Contents

A portion (0.5 g) of the samples was submerged in 1 mL of N,N-dimethyformamide at 4 °C for 16 h. The absorbance of chlorophylls *a* and *b* released in the solvent was measured at 663.8 nm and 646.8 nm. The contents of chlorophylls a and b were calculated using absorbance as follows: chlorophylls *a* and *b* (μM) = 19.4 × A_646.8_ + 8.05 × A_663.8_ [57,58].

### 4.5. Protein Extraction and Immunoblot Analysis

A portion (500 mg) of samples was ground with a mortar and pestle in 500 µL of RIPA extraction buffer (Nacalai Tesque, Tokyo, Japan), which contains 50 mM Tris-HCl, 150 mM NaCl, 1% Nonidet-P40, 0.1% sodium deoxycholate, 0.1% SDS, and protease inhibitor. The suspension was centrifuged twice at 16,000× *g* at 4 °C for 10 min, and the supernatant was used as a protein sample. Protein concentrations were measured for absorbance at 595 nm by the Bradford assay [59] using standardized protein solutions of bovine serum albumin.

SDS-sample buffer (Bio-Rad, Hercules, CA, USA), which contains 62.5 mM Tris-HCl (pH 6.8), 2% SDS, 50 mM dithiothreitol, 10% glycerol, and 0.01% bromophenol blue, was added to protein samples. Proteins (10 µg) were separated by electrophoresis on 10% polyacrylamide gel and stained with Coomassie brilliant blue as a loading control. On the other hand, proteins in the gel were transferred onto a PVDF membrane using a semidry transfer blotter. The blotted PVDF membrane was blocked for 5 min in Bullet Blocking One reagent (Nacalai Tesque). After blocking, the PVDF membrane was cross-reacted with the primary antibodies for 30 min. As the primary antibodies, anti-V ATPase (Agrisera, Vännäs, Sweden), ascorbate peroxidases [60], glutathione reductase (Agrisera), Cu/Zn superoxide dismutase (Proteintech, Rosemont, IL, USA), and peroxiredoxin [61] antibodies were used. Anti-rabbit IgG conjugated with horseradish peroxidase (Bio-Rad) was used as the secondary antibody. After incubation for 30 min, signals were detected using the 3,3′,5,5′-tetramethylbenzidine membrane peroxidase substrate system (SeraCare, Gaithersburg, MD, USA). The integrated densities of bands were calculated with ImageJ software (version 1.8; National Institutes of Health, Bethesda, MD, USA).

### 4.6. Assay of Ascorbate-Peroxidase Activity

Ascorbate peroxidase activity was determined by monitoring the oxidation of ascorbic acid at 290 nm (absorption coefficient ε = 2.8 mM^−1^ cm^−1^) [62]. A portion (250 mg) of samples was homogenized with 2.5 mL of 25 mM potassium phosphate buffer (pH 7.8), which contains 2% polyvinylpolypyrrolidone, 0.4 mM EDTA-4H, and 1 mM ascorbic acid. After centrifugation at 15,000× *g* at 4 °C for 20 min, the soluble fraction was filtered by a layer of miracloth, and the eluted solution was an enzyme crude extract. For a measurement of ascorbate-peroxidase activity, the assay medium comprised 25 mM potassium phosphate buffer (pH 7.0), 0.25 mM ascorbic acid, 0.4 mM EDTA-4H, and 0.1 mM hydrogen peroxide.

### 4.7. RNA Extraction, cDNA Synthesis, and PCR Analysis

Total RNA was isolated with the RNeasy Plant Mini Kit (Qiagen, Venlo, The Netherlands) according to the protocol of the manufacturer. A portion (500 mg) of samples was flash-frozen in liquid nitrogen and ground to a powder using a mortar and pestle. First-strand cDNA was synthesized from total RNA (1 μg) with the iSuperscript Reverse Transcription Supermix (BioRad). Gene-specific primers were constructed with Primer3Plus software [63] (https://www.bioinformatics.nl/cgi-bin/primer3plus/primer3plus.cgi/, accessed on 11 December 2024) and used to amplify the 200–500 bp regions. Gene-specific primers for *18S rRNA* (X02623) (F 5′-TGATTAACAGGGACAGTCGG-3′; R 5′-ACGGTATCTGATCGTCTTCG-3′) and *bet v1* (A0A3B6TLM4) (F 5′-ACAGCTGGACCCACGAGATC-3′; R 5′-CAGAATCCTTGGCCTTGGTA-3′) were synthesized. PCR analysis was performed with the Emerald Amp PCR Master Mix (Takara, Tokyo, Japan) as follows: at 98 °C for 10 s, at 60 °C for 30 s, and at 72 °C for 30 s, for a total of 30 cycles. The amplified products were separated on 3% agarose gel and stained with the Atlas Clear Sight Gold DNA stain (BioAtlas, Tartu, Estonia). The integrated density of bands was calculated with ImageJ software.

### 4.8. Hydrogen-Peroxide Content Measurement

Hydrogen peroxide contents were measured with the Amplite Colorimetric Hydrogen Peroxide Assay Kit (AAT Bioquest, Pleasanton, CA, USA). A portion (200 mg) of fresh roots was ground with a mortar and pestle in 200 μL of phosphate buffer on ice. The homogenates collected were centrifuged at 16,000× *g* for 20 min, and the supernatant was immediately used for the hydrogen peroxide assay. A working solution (25 µL) containing horseradish peroxidase and hydrogen peroxidase substrate was added to 25 µL of sample, which was then reacted in the dark. After 10 or 60 min of reaction, hydrogen peroxide contents were measured for absorbance at 650 nm with hydrogen peroxide as the standard and calculated from a standard curve.

### 4.9. Statistical Analysis

The statistical significance of the 2 groups was evaluated by Student’s *t*-test and a *p*-value of less than 0.05 was considered statistically significant.

## 5. Conclusions

Soybean is in high demand as a food material due to being rich in protein and functional ingredients; however, it is vulnerable to environmental stress. On the other hand, soybean growth under salt stress can be promoted by the application of TiO_2_ NPs. To further enhance the growth-promoting effect of TiO_2_ NPs on soybeans, they were biologically synthesized. Main findings on the effect of GS-TiO_2_ NPs are as follows: by additional treatment with GS-TiO_2_ NPs, (i) root elongation of soybean suppressed by salt stress was restored to the control level; (ii) the abundance of V-ATPase decreased in roots under salt stress was recovered; (iii) the abundance of glutathione reductase increased in roots and hypocotyls by salt stress was recovered; and (iv) hydrogen-peroxide production upregulated in roots by salt stress was restored. These results suggest that GS-TiO_2_ NPs may promote soybean growth by detoxifying hydrogen peroxide, which increases under salt stress, by upregulating the ROS scavenging system.

## Data Availability

All data are contained in this article and in Supplementary Materials.

## Supplementary Materials

The following supporting information can be downloaded at: https://www.mdpi.com/article/10.3390/ijms26178309/s1.

## Abbreviations

The following abbreviations are used in this manuscript:

GS	green synthesized
CS	chemical synthesized
NPs	nanoparticles
FTIR	Fourier transform infrared
PCR	polymerase chain reaction
ROS	reactive oxygen species
PR10	pathogenesis-related 10
MLPs	major latex proteins
