# Supplementary material for: Green Synthesized Titanium Oxide Nanoparticles Promote Salt Tolerance in Soybean
Source: Int J Mol Sci. 2025 Aug 27;26(17):8309. doi: 10.3390/ijms26178309 (PMC12428493; doi:10.3390/ijms26178309)
Supplement: Supplementary file 1 [file ijms-26-08309-s001.zip › ijms-3731956-supplementary.pdf]

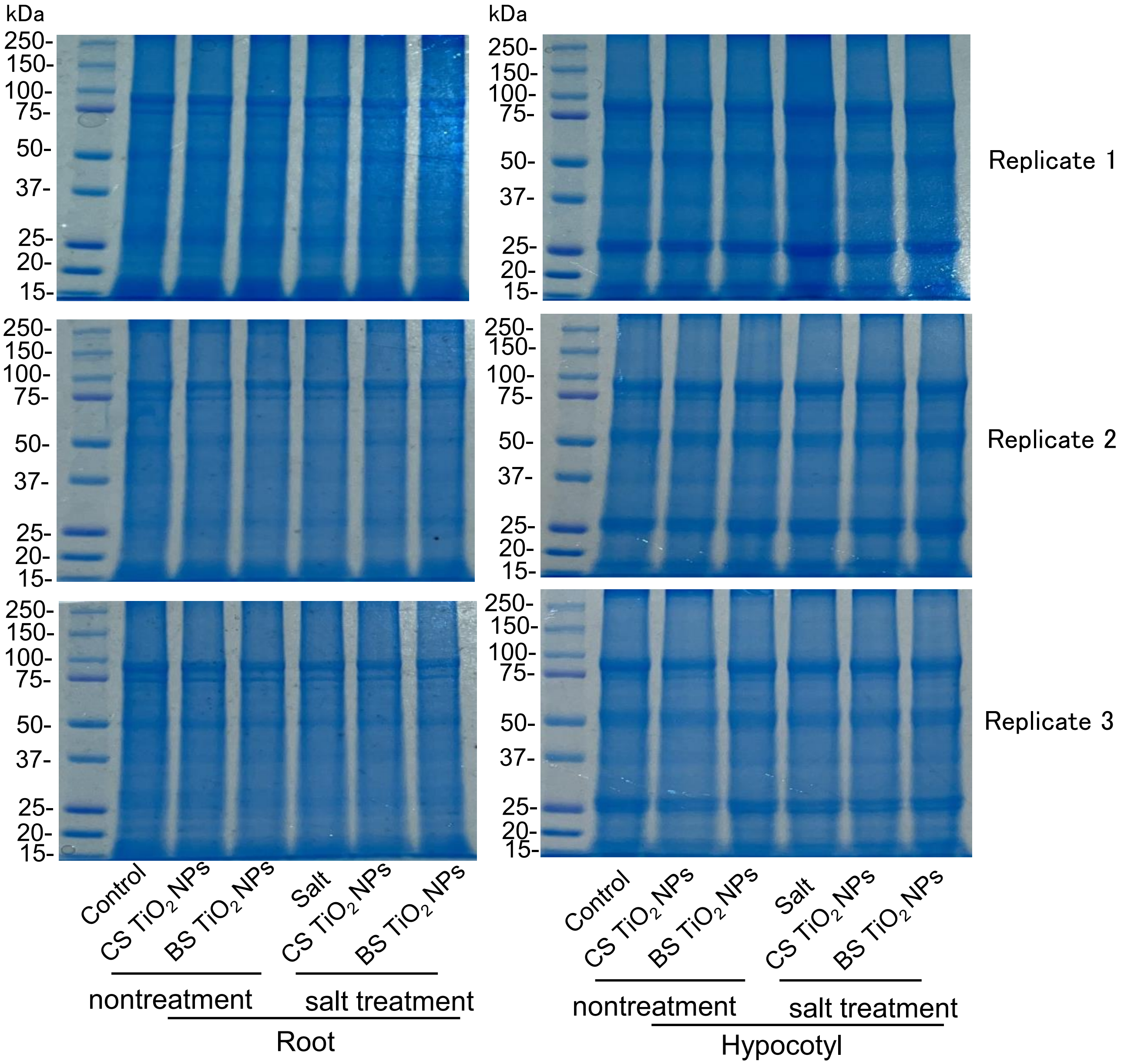

Figure S1. The Coomassie-brilliant blue staining pattern of proteins used for immunoblot analysis.

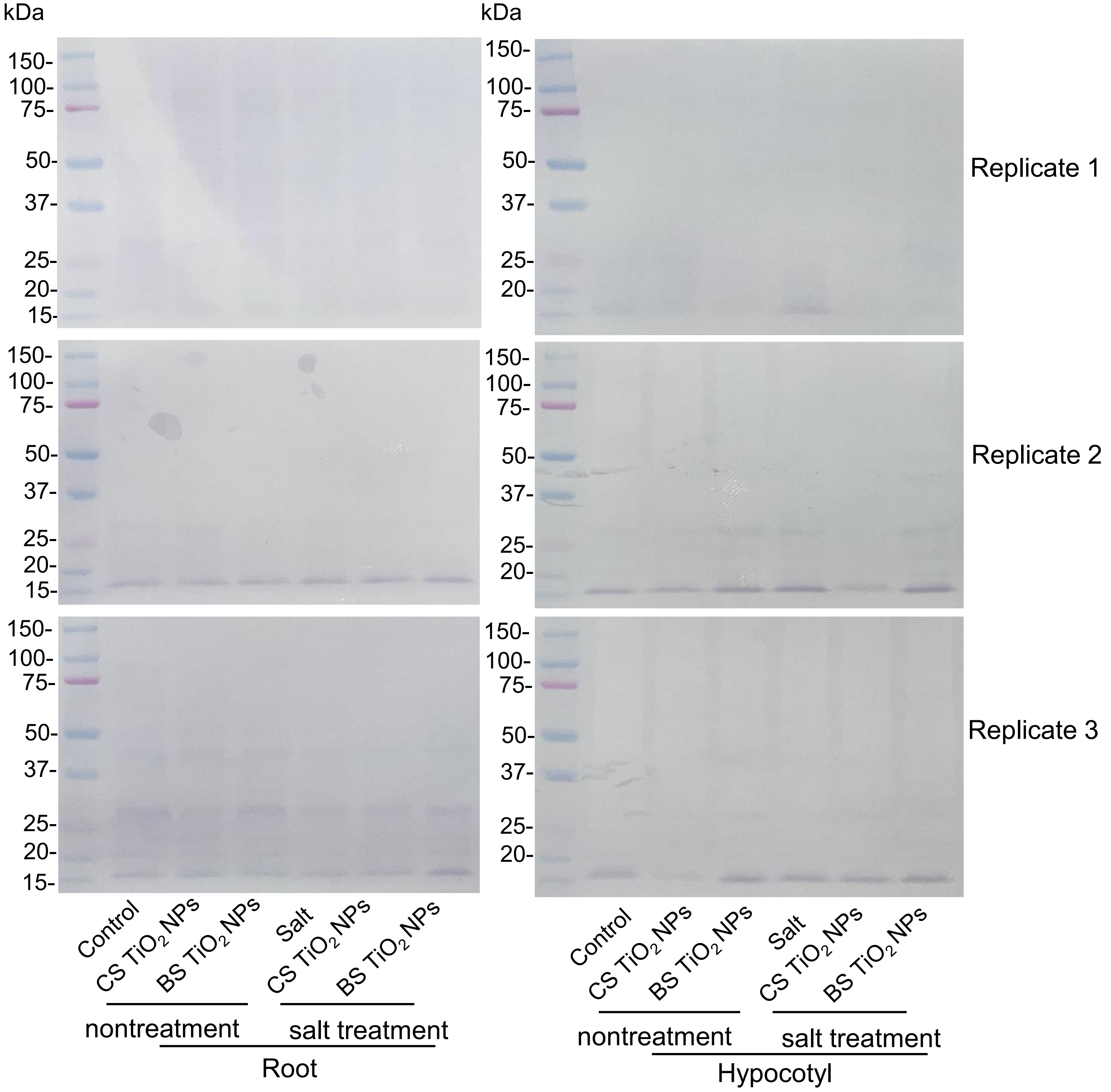

Figure S2. Blots of the entire membrane with anti-superoxide dismutase antibody, which were used in Figure 6A.

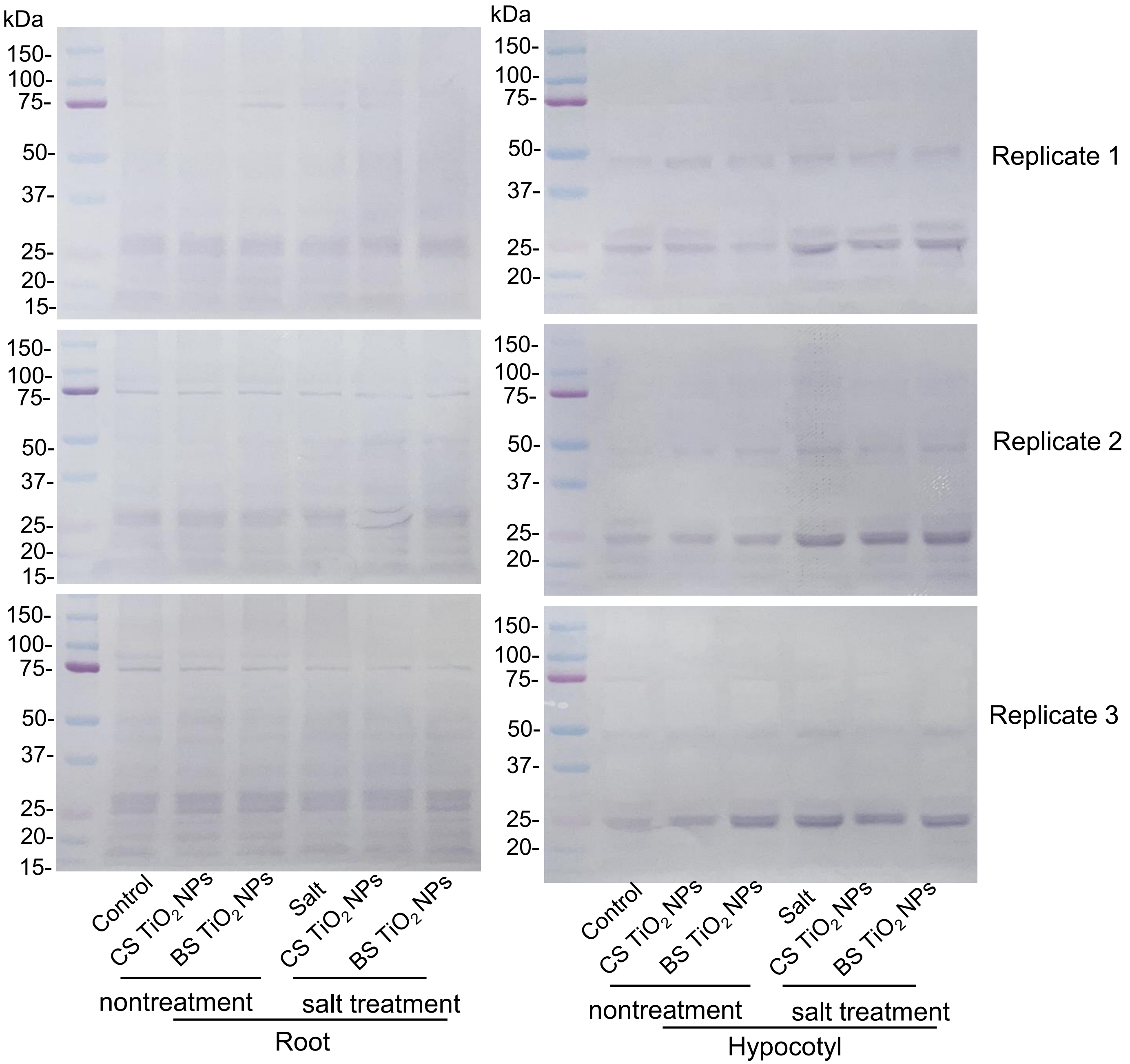

Figure S3. Blots of the entire membrane with anti-peroxiredoxin antibody, which were used in Figure 6B.

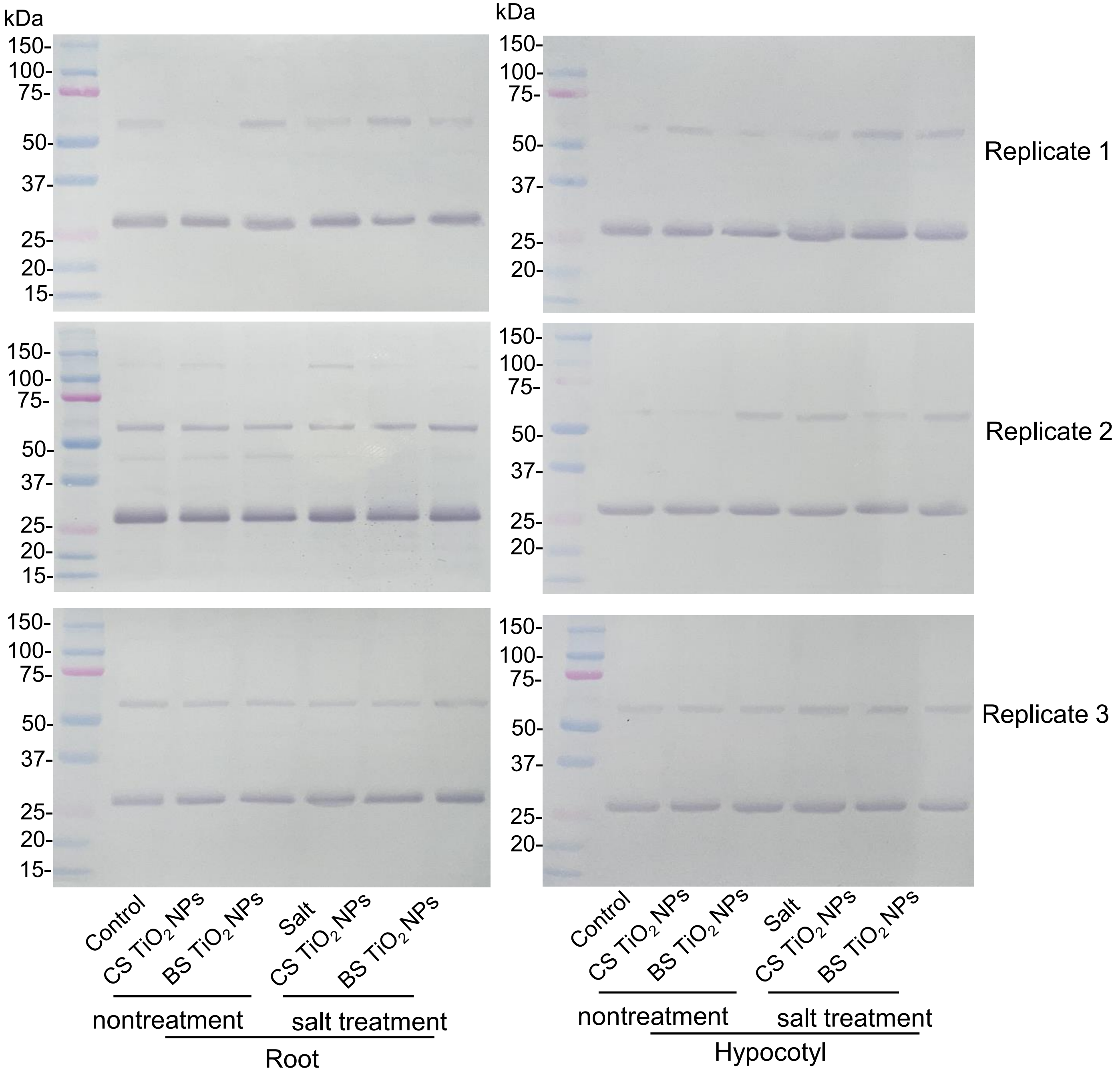

Figure S4. Blots of the entire membrane with anti-ascorbate peroxidase antibody, which were used in Figure 6C.

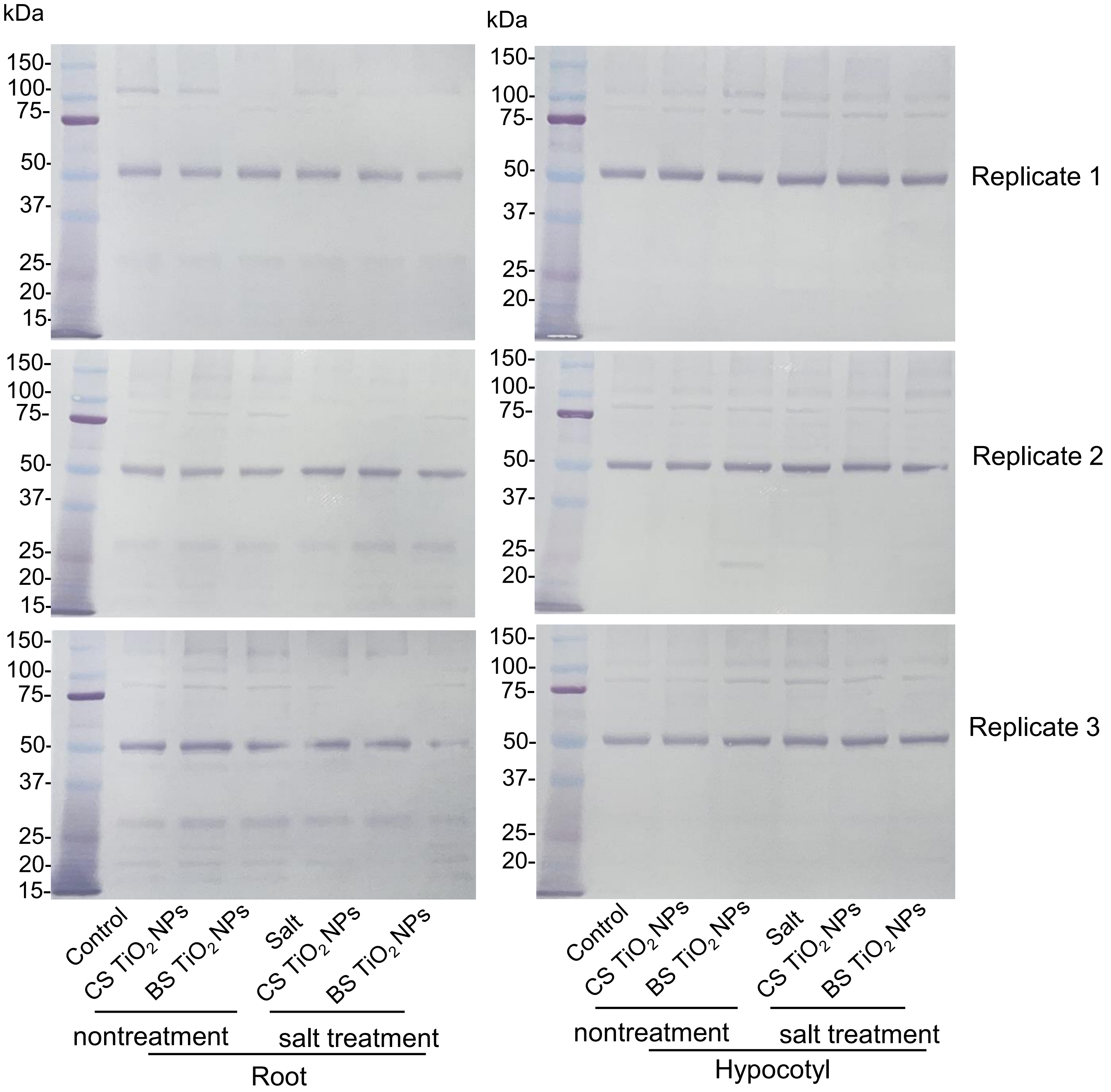

Figure S5. Blots of the entire membrane with anti-glutathione reductase antibody, which were used in Figure 6D.

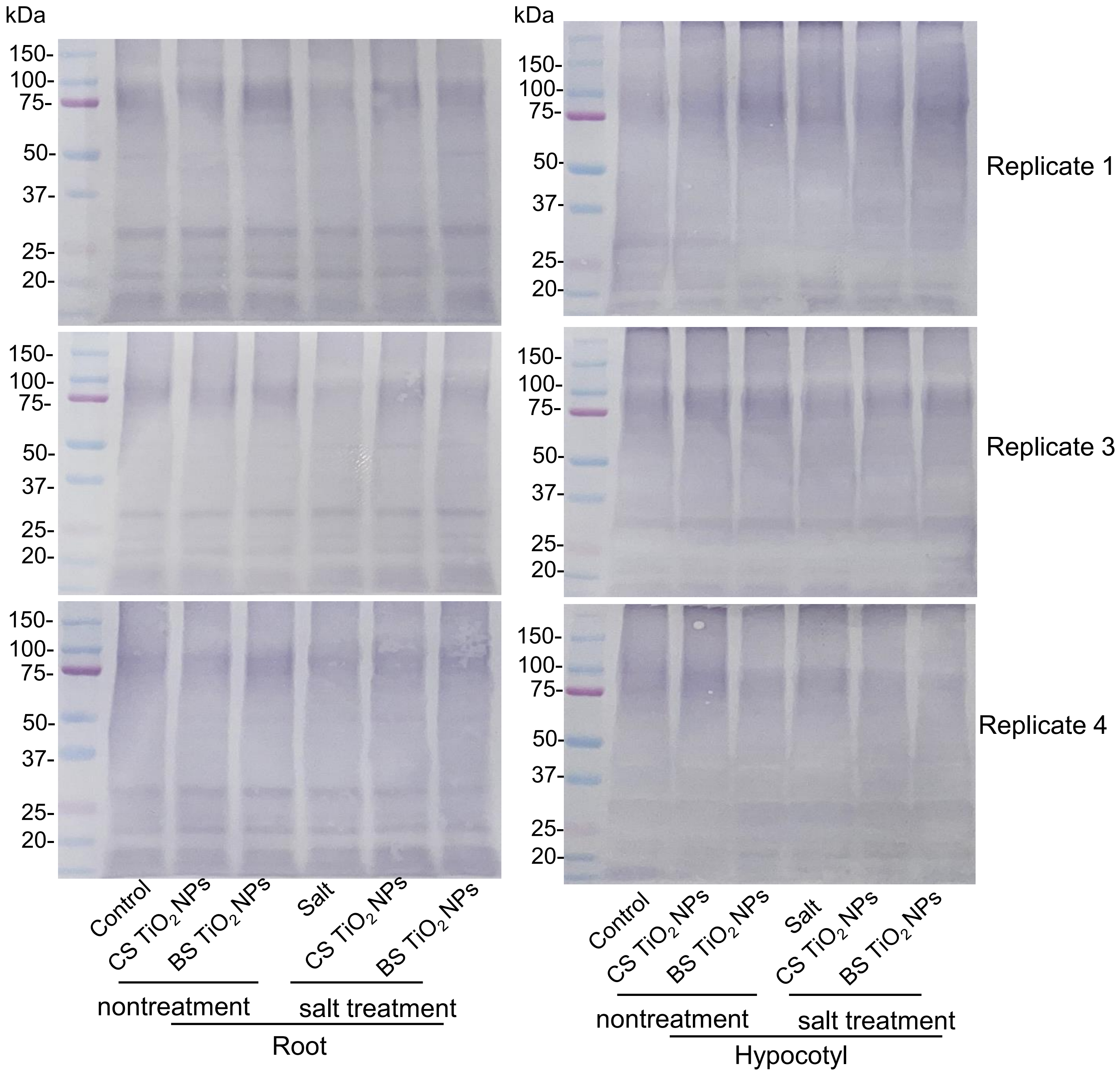

Figure S6. Blots of the entire membrane with anti-V ATPase antibody, which were used in Figure 7.
